# Role of microbiota and microbiota‐derived short‐chain fatty acids in PDAC

**DOI:** 10.1002/cam4.5323

**Published:** 2022-10-07

**Authors:** Hülya Yılmaz Temel, Öznur Kaymak, Seren Kaplan, Basak Bahcivanci, Georgios V. Gkoutos, Animesh Acharjee

**Affiliations:** ^1^ Department of Bioengineering, Faculty of Engineering Ege University Izmir Turkey; ^2^ Institute of Cancer and Genomic Sciences, University of Birmingham Birmingham UK; ^3^ National Institute for Health Research Surgical Reconstruction, Queen Elizabeth Hospital Birmingham Birmingham UK; ^4^ MRC Health Data Research UK (HDR UK) Birmingham UK

**Keywords:** host–microbe interactions, inflammation, microbiome, microbiota, pancreatic ductal adenocarcinoma, SCFA

## Abstract

Pancreatic ductal adenocarcinoma (PDAC) is one of the most aggressive lethal diseases among other cancer types. Gut microbiome and its metabolic regulation play a crucial role in PDAC. Metabolic regulation in the gut is a complex process that involves microbiome and microbiome‐derived short‐chain fatty acids (SCFAs). SCFAs regulate inflammation, as well as lipid and glucose metabolism, through different pathways. This review aims to summarize recent developments in PDAC in the context of gut and oral microbiota and their associations with short‐chain fatty acid (SCFA). In addition to this, we discuss possible therapeutic applications using microbiota in PDAC.

## Graphical abstract

Gut microbiome and its metabolic regulation play a crucial role in PDAC. Metabolic regulation in the gut is a complex process that involves diet intake, microbiome and microbiome‐derived short‐chain fatty acids.

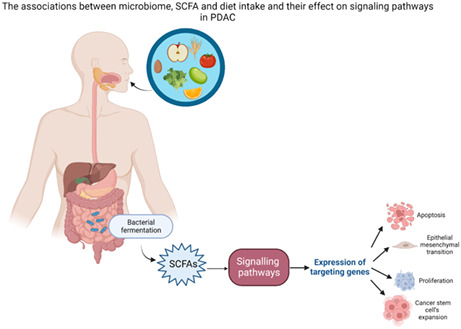

## 1 INTRODUCTION

Pancreatic ductal adenocarcinoma (PDAC) is one of the most aggressive cancer types causing oncologic mortality.^1^ The majority of patients are diagnosed at an advanced stage with a poor prognosis; only 9% of patients exceed 5‐year survival.^2^ PDAC accounts for more than 90% of all pancreatic cancers and is estimated to become the second leading cause of cancer death by 2030.^3^

Surgery and cytotoxic chemotherapy and radiation therapy form the standard treatment options for PDAC. When pancreatic surgery is combined with adjuvant and/or neoadjuvant treatment, the patient's long‐term survival and quality of life can be improved. Pancreatic resection is one of the most challenging and risky abdominal surgery type due to the high risk of complications.^4^, ^5^ Furthermore, less than 20% of pancreatic cancer patients are surgically resectable, primarily due to their particular metastatic state.^5^, ^6^ Therefore, there is a crucial need for the identification of early‐stage detection as well as for innovative and more effective therapies.

The role of microbiome and its implication in cancer treatment is an emerging area. According to the increasing number of preclinical and clinical studies, gut microbiota can potentially play a role in the therapeutic potential of cancer patients as well as in their response to immunotherapy and chemotherapy.^7^

Indigestible carbohydrates (e.g., dietary fibers) are fermented by gut microbiota and acetate, propionate and butyrate are produced. Acetate's molar ratio is three times higher than butyrate and propionate.^8^ But this proportion can change according to diet, site of fermentation and host genotype.^9^ Colonocytes utilize most of the butyrate as an energy source.^10^ SCFAs enter the liver through the portal vein^11^ and propionate and acetate are metabolized to generate glucose and used as a substrate in lipogenesis.^10^, ^12^ Due to SCFAs capacity to reach different systematic tissues, they also have effect on regulating immune system, anti‐inflammatory response, blood pressure and energy intake.^13^, ^14^

SCFAs are G‐protein coupled receptors (GPCR) 41 and 43 ligands. Given GPR41 and GPR43 are expressed across a number of different tissues such adipose, intestinal and skeletal muscle, pancreatic and liver tissues,^10^, ^15^, ^16^ SCFAs have an important role in the peripheral tissues and gut function.^10^

Herein we highlight recent developments of microbiota involvement in PDAC and their associations with short‐chain fatty acids (SCFAs).

### 1.1 Microbiome in PDAC

The human microbiota is made up of 10–100 trillion microbial cells which are living symbiotically influenced by a number of factors including genetic variation, environment, and diet. The microbiome plays a crucial role in maintaining homeostasis and dysbiosis,which can influence the pathogenesis of many diseases,^17^ as well as tumor response to therapies.^18^

In recent studies, the evidence of bacterial and fungal populations in normal pancreatic tissue and PDAC samples were shown and it was reported that the microbiome of PDAC samples are different from healthy samples.^19^, ^20^, ^21^ Moreover, it has been recently reported that tongue coating microbiota diversity in PDAC patients is significantly high, and bacterial composition is different between healthy people and PDAC patients.^22^

The association between the PDAC and microbiota was first defined by a study on pancreatitis patients determining *H. pylori* ^23^, ^24^ implication, which is now accepted as a risk factor for PDAC.^16^ Since then several studies were published discussing diverse microbiota alterations, including ones occurring in oral, pancreatic, and gastrointestinal tissues,^25^, ^26^ biopsy, blood, stool, salivary, and oral swab samples by 16S ribosomal RNA (16S rRNA) sequencing.^25^

In recent years it has been indicated that the gut microbiome and its metabolites are closely related with human health and disease highlighting important questions such as whether the interaction of the gut microbiome and associated metabolites lead to particular diseases and whether particular diseases affect the gut microbiome alteration and SCFAs.

Associations between the gut microbiome and complex traits have been identified by microbiome‐wide association studies for a number of different diseases, such as obesity and type 2 diabetes,^27^ but whether these associations form causal relationships remain to be understood. In 2019, Sanna et al. assembled genome‐wide genetic data, fecal SCFA measurements, gut metagenomic sequencing data clinical phenotypes and also collected publically available genome‐wide association summary statistics for glysemic and anthropometric traits. It was reported that, gut‐produced SCFAs, especially propionate and butyrate have causal role in terms of energy balance and glucose homeostasis in man.^28^

Several studies have depicted that the microbiota diversity and alterations can be associated with PDAC initiation and progression.^24^, ^29^, ^30^, ^31^, ^32^

In one such study fecal samples from 85 PDAC patients and 57 healthy controls were collected and analyzed for microbial characteristics reporting that gut microbial diversity was significantly lower in PDAC patients. PDAC patients' gut microbiota contained significantly higher Bacterioidetes and lower firmicutes and Proteobacteria compared with healthy controls.^29^ In another study, the bacterial composition of pancreatic fluid, bile and jenunal fluid, fecal samples were characterized in 50 patients undergoing pancreaticoduodenectomy denoting that the microbial diversity in fecal samples was significantly lower than healthy samples mainly enriched with Klebsiella and Bacteroides.^33^ Half et al. (2019) analyzed fecal microbiota of 30 PDAC patients, 13 health individuals and 16 individuals with non‐alcoholic fatty liver disease. No difference in microbial diversity was depicted between groups, but it was reported that PDAC patients had distinct microbial profile compared with control group.^34^ In 2020, Kohi et al. analyzed fungal and bacterial profiles of duodenal fluid from 74 PDAC patients, 98 pancreatic cysts patients and 134 normal individuals reporting that, PDAC patients had significantly decreased fungal and bacterial diversity when compared to others who had pancreatic cysts and health individuals. There was not any significant difference between the duodenal fluid microbiota profiles of patients with pancreatic cysts and healthy individuals.^35^ Finally, more recently, Guo et al. (2022) reported that microbiome variation is related to pancreatic cancer.^36^

### 1.2 Gut and oral microbiota

The human gut microbiota form a diversified ecosystem that caters the defense against digestion anomalies, and infections, as well as contributes to the adjustment of gut hormone secretion and the immune system regulation.^37^ The disturbances of the GI system microbiota could bring information about pathology, mainly diseases linked to metabolism and autoimmunity. Several studies have focused on the role of microbiota on carcinogenesis, especially the potential association of gut microbiota and colorectal cancer.

Some studies demonstrate the importance of gut microbiota in PDAC by analyzing characteristics of microbial communities of pancreatic cancer patients and the microbiome diversity of healthy controls. Ren et al. (2017) described that gut microbial diversity and alpha diversity are decreasing in pancreatic carcinoma in terms of the microbial profiling of mainly *Prevotella, Veillonella, Klebsiella, Selenomonas, Hallella, Enterobacter, Cronobacter, Gemmiger, Bifidobacterium, Coprococcus, Clostridium IV, Blautia, Flavonifractor, Anaerostipes, Butyricicoccus, Dorea*. On the other hand these traits play a role in the considerable increase of some potential pathogens and lipopolysaccharides (LPS)‐producing bacteria.^29^ Abdul Rahman et al. (2021) indicates that the gut microbiota in humans comprises mainly four phyla: *Bacteriodetes, Firmicutes, Proteobacteria,* and *Actinobacteria*.^38^

Pushalkar et al. (2018) collected fecal samples from PDAC patients with stage I/II and stage IV and compared them with healthy controls reporting a significant difference between patients in stage I/II and stage IV. While Phascolarctobacterium, Alcaligenaceae, Paraprevotellaceae, and Synergistaceae were present in high abundance in stage I and II PDAC, Veillonella, and Streptococcus were detected in high presence in patients with stage IV PDAC.^20^ In 2019, Del Castillo et al. reported that, the relative abundances of Fusobacterium, Porphyromonas, Capnocytophaga, idocharacter, Prevotella, Gemella and Selemonas was higher in pancreatic cancer patients when compared with healthy people.^39^ The changes in microbiome in vivo are highly correlated with in cancer patients and cancer progression.^36^ Future human studies are necessary to fully understand the effect of microbiome in different periods of pancreatic cancer.

Although immunotherapy in PDAC patients is not effective, recent studies denote that the characteristics of gut microbiota adjust /modulate immunotherapy response effecting its effectiveness.^40^, ^41^, ^42^, ^43^

More than 700 varied microorganisms colonized in the oral cavity with *Porphyromonas gingivalis* and *Aggregatibacter actinomycetemcomitans* being abundant in PDAC.^44^ In another study, Farrell et al. (2012) investigated the variations of salivary microbiota and evaluated their potential associations with pancreatic cancer and chronic pancreatitis. This study reported a significant level of abundances of salivary microflora: *Neisseria elongate* and *Streptococcus mitis* which are low in PDAC compared to healthy controls.^45^ Together with periodontal pathogen *P. gingivalis*, Fusobacterium (anaerobic, gram‐negative oral bacterium) strains are found and treated as a pathogen.^45^ Nevertheless, in several large cohort studies, Fusobacterium has been reported to be behaving differently, that is, reducing PDAC risk.^25^, ^44^ Despite these conflicting results, Kostic et al. (2013) reported that *Fusobacterium* potentiates tumorigenesis and Wei et al. (2019) also reported that reactive oxygen species (ROS) and inflammatory cytokines production could be increased by *Fusobacterium*, and *Fusobacterium* attenuate the tumor immune microenvironment and drive myeloid cell infiltration in intestinal tumors.^25^, ^46^ Several other microbiome abundances have been reported, such as *Aggregatibacter* (lower abundance), *Corynebacterium* (lower abundance), *Granulicatella adiacens* (higher abundance), Bacteroides (higher abundance).^25^

## 2 THE ROLE OF HUMAN PANCREAS AND INTRAPANCREATIC MICROBIOTA IN PDAC

The intestinal bacteria are essential for the pancreas as they are necessary for the breakdown of hydrolytic enzymes secreted through the pancreas. Moreover, the pancreatic juice's antibacterial activity in the human pancreas might protect the pancreatic tissue from retrograde infections.^25^ Fritz et al. (2010) and Pushalkar et al. (2018) indicated that the base of the potential etiological roles of gut microbes in pancreatic cancer may exist because they are able to reach the pancreas by biliary/pancreatic duct or the circulatory system.^20^, ^47^ The system of pancreatic carcinogenesis and its link between microbial flora should be well understood because *H. pylori* may not directly take part in triggering pancreatic carcinogenesis. For instance, Jesnowski et al. (2010) indicate that *H. pylori* leads to gastric lesions by precisely impairing the human gastric mucosa. Although its own DNA can be identified in infected corpus stomach tissues and antrum,^48^ it is undetectable pancreatic juice. Furthermore, Jesnowski et al. (2010), based on a chronic pancreatitis study, suggested that this bacterium may not influence the disease in a direct way.^49^ Such studies motivated researchers to investigate other indirect mechanisms, such as immune escape, inflammation, and exhibition of carcinogenic nitrosamines, which should also be taken into consideration as a fundamental mechanism.

### 2.1 Potential novel therapy strategies in PDAC using microbiome

#### 2.1.1 Probiotics and prebiotics

Dietary prebiotics (e.g., non‐digestible oligosaccharides fructans and galactans) are metabolized by either bifidobacteria^50^ or by host microorganisms that can easily utilize and convert them into metabolic products, such as butyrate, acetate, and propionate. These metabolic products are critical to gut health,^51^ and beneficial to human health.^52^ Up to date, there are no reports that have been published on the potential relations between prebiotics and PDAC. However, Abdul Rahman et al. (2021) reported that prebiotics might act in a very probiotic‐independent direct manner.^38^ Moreover, several studies have indicated probiotics having a positive effect in suppressing tumorigenesis through partaking within the natural resistant framework. Furthermore, probiotics have been associated with diminishing oxidative stress, progressing the community of enteric microbiota, improving intestinal boundary work, and balancing colonization of the pathogenic bacteria.^38^, ^53^, ^54^, ^55^ Van Minnen et al. (2007) used a rat model to explore the effects of multispecies probiotics *(Lactobacillus casei W56, Lactobacillus acidophilus W70, Lactococcus lactis W58, Lactobacillus salivarius W24, Bifidobacterium infantis W52 and Bifidobacterium bifidum W23)* and investigated whether the modulation of the intestinal flora by probiotics could decrease bacterial translocation.^56^ In another study, Akyol et al. (2003) evaluated the effects of *Saccharomyces boulardii* as well as used a combination of two antibiotics (meropenem and ciprofloxacin) using an acute pancreatitis mice model.^57^ Oláh et al. (2002) used the first human model to research the probiotics' effects (*Lactobacillus plantarum 299*) for pancreatitis treatment, reporting that this bacillus had no side effects and was helpful in lowering pancreatic sepsis and surgical interventions. This study also demonstrated that lactic acid bacteria in the gut have a part in carcinogenesis regression due to their impact on immunomodulation.^58^ Five years later, Oláh et al. (2007) reported that early nasojenual feeding with synbiotics (a mixture of probiotics and prebiotics) may inhibit organ dysfunctions in the late phase of severe acute pancreatitis.^59^ Górska et al. (2019) indicated that probiotic bacteria have the potential to both boost and reduce the production of anti‐inflammatory cytokines, which play a vital role in the prevention of carcinogenesis, depicting that *Bacillus polyfermenticus and Lactococcus lactis* are decreasing the cancer cell proliferation in both colon cancer and human gastric adenocarcinoma.^60^ Lutgendorff et al. (2008) highlighted probiotics' boosting effect on pancreatic glutathione biosynthesis as well as their effect in decreasing oxidative stress in experimental acute pancreatitis.^61^ Chen et al. (2020) investigated the effects of probiotics as an adjuvant for pancreatic cancer during chemotherapy. PDAC mice models were employed to study the influence of multi‐strain probiotics (*Lactobacillus reuteri* GMNL‐89 and *Lactobacillus paracasei* GMNL‐133) and a combination treatment with gemcitabine and probiotics. The study concluded that the inclusion of probiotics as an adjuvant or combination therapy should be considered viable therapeutic strategies.^62^

#### 2.1.2 Synbiotics and postbiotics

Not much is known about the use and benefits of synbiotics and postbiotics in PDAC. Rad et al. (2021) reported that postbiotics, due to their antioxidant, anti‐proliferative, anti‐inflammatory, and anti‐cancer properties, modify the composition of the gut microbiota and influence the immune system's PDAC related activity. The study further depicted the postbiotics anti‐cancer potential by highlighting the postbiotic strain *Lactobacillus acidophilus*' antiproliferative effect in pancreatic cancer patients in line with the postbiotics' cancer treatment potential reported by Vrzáčková et al. (2021).^63^, ^64^ SCFAs (acetate, propionate, and butyrate) are some of the most investigated and well‐known postbiotics. SCFAs are produced by gut microbes, primarily *Faecalibacterium prausnitzii* and *Eubacterium rectale*, during the fermentation of dietary fiber.^64^

#### 2.1.3 Antibiotics and fecal microbiota transplantation (FMT)

Quinolones, nitroimidazoles, beta‐lactams, tetracyclines, glycopeptides, and macrolides are the most commonly used antibiotics.^65^ PDAC‐antibiotics association studies have revealed that gut microbiota induce an immunogenic re‐programming process of the microenvironment of tumor, as well as suppress tumor growth by inducing anti‐tumourigenic T‐cell activation. They further help to boost immune response and improve immunotherapy sensitivity. Such capabilities offer the potential of being part of potential PDAC therapeutics approaches. Mohindroo et al. (2019) reported that the use of macrolide antibiotics, for more than 3 days, during treatment, resulted in a longer progression‐free survival (PFS) and overall survival (OS) based on a retrospective analysis across 148 patients with metastatic PDAC.^66^ On the other hand, some studies have reported potential antibiotics association with shorter overall survival. Hasanov et al. (2019) reported that tetracycline use was substantially related to shorter survival in patients with resected PDAC when compared to the other antibiotics (quinolones, beta‐lactams, nitroimidazoles, glycopeptides, macrolides) utilized as well as depicted a trend toward a shorter PFS in patients with resectable PDAC.^65^ Using a PDAC‐bearing Pdx1Cre;LSL‐KrasG12D;Trp53R172H (KPC) mice model, Pushalkar et al. (2018) employed antibiotics and showed bacterial ablation, reporting an anti‐tumor influence that could be reversed, with fecal transferation from PDAC mice, whereas a fecal transferation from non‐PDAC controls had no effect.^20^ Fecal microbiota transplantation (FMT), which includes more bacteria than regularly used probiotic supplements, represents a potential strategy to overcome immunosuppression and resistance to treatment in cancer patients with a low chance of survival.^7^, ^67^ Riquelme et al. (2019) found that when FMT was used in an animal model treated with antibiotics, the intestinal flora colonized pancreatic tumors and altered the overall bacterial composition within the tumor.^68^ Animal studies have also shown a protective effect of gut and tumor bacteria in PDAC patients (long‐term survivors) who survived for more than 5 years without signs of illness.^7^

### 2.2 Drug resistance of the microbiome

Drug resistance also plays a role on the microbiome. For example, Gemcitabine, is a chemotherapy drug that is used in patients with pancreatic cancer. Additionally, *Gammaproteobacteria* were found in PDAC tissue specimens with gemcitabine resistance by Geller et al. (2017), who hypothesized that this type of bacteria could regulate tumor sensitivity to gemcitabine.^38^ In terms of resistance mechanisms, Quiñonero et al. (2019) analyzed the different mechanisms of genetic and protein resistance by which PDAC cells reduce the effectiveness of available drugs. They indicate that resistance is accomplished by different mechanisms, such as mutations in genes involved in important metabolic pathways and non‐coding RNAs (ncRNAs) that regulate the expression of genes involved in cellular behavior. On the other hand, PDAC cancer stem cells (CSCs) have direct drug resistance effect due to their capability in overexpression of ABC genes, aldehyde dehydrogenase enzymes and poly (ADP‐ribose) polymerases. ABC genes are involved in drug transport, aldehyde dehydrogenases are affined in cellular drug metabolism and poly (ADP‐ribose) polymerases play a crucial role in drug‐induced DNA damage repair.^69^ Antibacterial exposure, however, was linked to an increased risk of gemcitabine‐linked toxicity during and after antibiotic exposure in the MPACT clinical trial involving 430 patients with metastatic PDAC treated with first‐line gemcitabine on the comparator arm (hazard ratio [HR]: 1.77; CI: 1.46–2.14).^38^, ^70^ Jia and Xie (2015) concluded that overcoming gemcitabine resistance presents several challenges. First, the understanding of the gemcitabine resistance mechanisms is still limited. Due to their interplay across a number of signaling pathways, inhibiting a particular signaling pathway is unlikely to result in a significant improvement in gemcitabine resistance. Second, despite promising results reported across a number of tumor and disease models, it is still too early to denote whether any of the identified drugs precisely target the developmental pathway in an effective and safe manner. Lastly, more research is needed to confirm the relevance of these pathways to gemcitabine resistance and to find a suitable treatment combination.^71^ A list of the microbiome up or down regulation is provided in the Appendix S1.

### 2.3 Microbiome and their role in the inflammation in the PDAC

Resident microbiota species contribute to the host immune system.^72^ Bacterial translocations may also occur due to interactions between organs. Data from microbial studies to date support the existence of sustained interactions between the mouth, gut, and pancreatic microbiomes. In particular, the result of the disruption of the gut microbiome is thought to be related to PDAC through a bacterial translocation and activation of various signaling pathways.^7^, ^68^, ^73^ Disruption of the microbiota is effective in tumor formation and growth. As a result of the induction of various chronic inflammatory reactions, due to the deterioration of the microbiota, a continuous infiltrating flow of metabolites and microorganisms can be observed.^24^ Furthermore, inflammatory cytokines and angionic markers are involved in PDAC development and progression.^74^ Cytokines, chemokines, reactive oxygen species (ROS), and bioactive small peptides are involved in the formation of local inflammation in the tumor microenvironment and are derived from infiltrating inflammatory cells, while bioactive small peptides may result from the degradation of proteins by tumor‐derived proteases.^75^ The PDAC exists in a microenvironment which includes mast cells, fibroblasts, T‐cells, neutrophils, macrophages, monocytes, and suppressor cells of myeloid origin, and various cytokines produced by these cells and by the tumor.^76^

Exposure to oxidative stress imbalance increases with the contribution of dysbiosis and microenvironmental inflammation. Sustained oxidative stress also increases apoptotic signals and triggers chronic inflammation, leading to cancer.^24^ In addition, microorganisms identified in PDAC produce immune tolerance by activating TLRs. TLR activation inhibits tumor growth by inhibiting apoptosis, and contributes to angiogenesis, rendering it easier for tumor tissues to reach their oxygen and nutrient needs, supporting and accelerating tumor development. In particular, TLR4 is overexpressed in human PDAC. Such overexpression is thought to accelerate cancer development.^25^, ^77^ Moreover, miRNA regulation, as a result of microbial changes, can also modulate host responses in pancreatic tissue by altering gene expression, while bacteria or pathogens that cause dysbiosis can also interfere by regulating miRNA expression.^73^ Depending on the microbial diversity and the presence of the dominant microbiome profile, some microorganisms affect tumor development in pancreatic cancer and others support the immune response of the host by exhibiting an anti‐tumor behavior. For example, the release and activation of CD8+ T cells in the presence of *Saccharopolyspora, Pseudoxanthomonas,* and *Streptomyces* strains support the anti‐tumor response.^68^ It was reported that gastric *H. pylori* and other enteric *Helicobacter* species were associated with samples of patients with pancreatic cancer, and 16S ribosomal DNAs were detected in the majority of pancreatic patients in the study.^78^

## 3 MICROBIOME AND ASSOCIATION WITH SCFA IN THE PDAC

### 3.1 The role of SCFA in cancer in general

The host genotype, a variety of environmental factors, and microbiota play a crucial role in cancer development.^24^, ^79^ There have been many studies aimed to identify the interactions between the gut microbiota and the host physiology.^80^ The variation in composition and diversity of the microbial community in the gut is directly linked with the cardiovascular function,^80^, ^81^ renal function,^80^, ^82^, ^83^ atherosclerosis,^84^ irritable bowel syndrome,^85^ and immune disorders.^86^, ^87^ Drug efficacy and safety is associated to the diversity of the gut microbiome potentially rendering as a viable feature of personalized treatments.^88^

Intestinal microorganisms generate SCFAs, such as acetate, propionate, and butyrate which are fermentation products.^89^, ^90^ The concentration of these SCFAs is mainly affected by diet and intestinal microbiota.^9^, ^91^ Other factors that affect the rate and amount of SCFAs generated include the colon pH.^51^, ^92^ Butyrate, acetate, and propionate have been reported to have some of the highest concentrations in colon,^93^, ^94^ whereas iso‐Butyric (C4), valeric (C5), and iso‐Valeric (C5) have some of lower ones.^94^, ^95^ Increased incidence of cancer and inflammatory diseases is related to poor fiber diets that affect SCFAs concentrations especially in breast and gastric cancers.^90^, ^95^, ^96^ SCFAs inhibit cell growth and migration, suppress histone deacetylase, and induce apoptosis to block and treat gastrointestinal and lung cancers.^90^, ^97^, ^98^, ^99^ The regulation of gut microbiota directly or indirectly effects the SCFAs concentration^90^ which can in turn provide viable cancer treatment strategies.^100^

**FIGURE 1:**
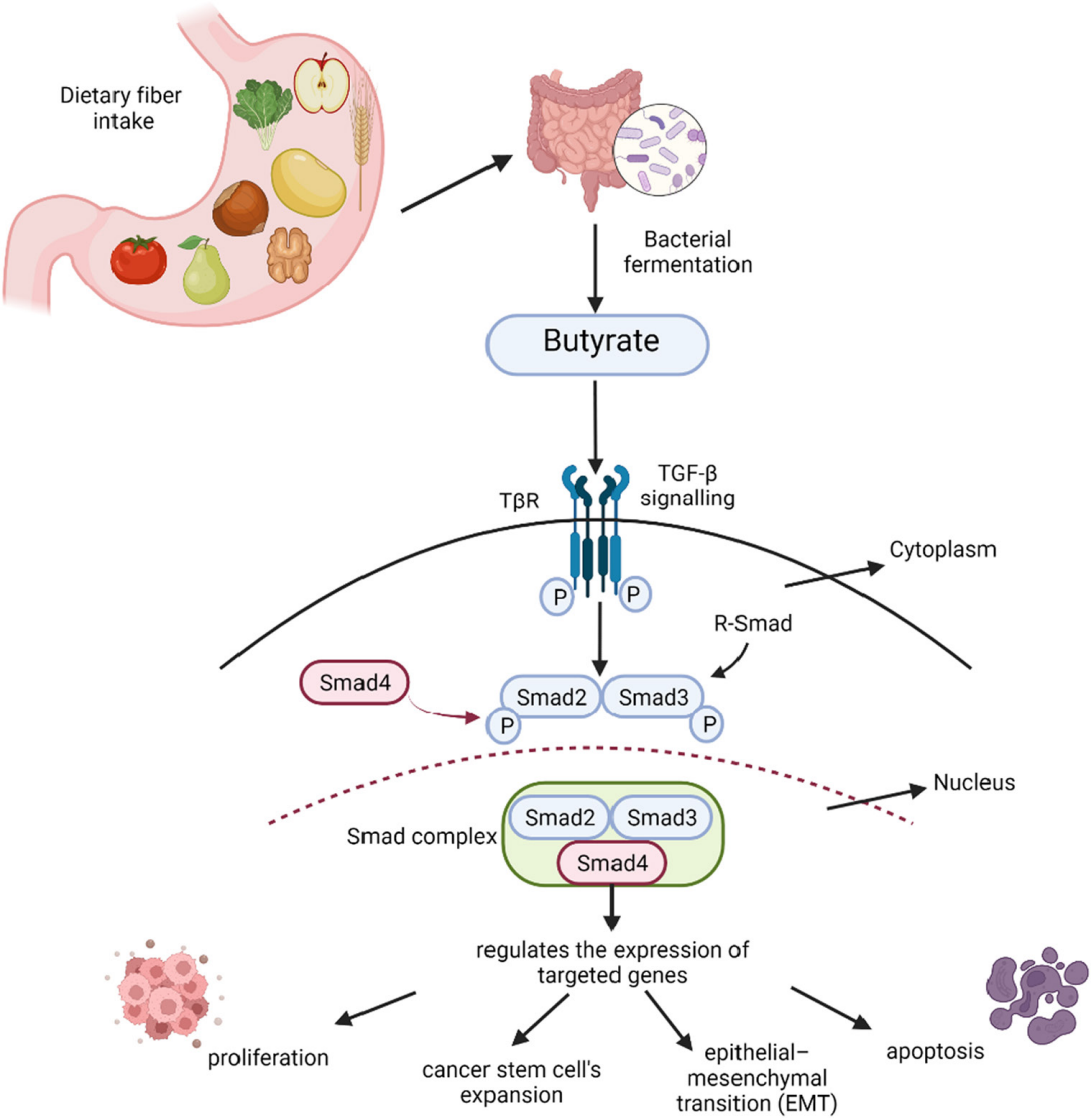
A graphical depiction of the associations between microbiome, SCFA and diet intake, and their effect on signaling pathways.

The association between microbiome and SCFA and their effect on signaling pathways is illustrated in Figure 1. The interaction between SCFAs and TGF‐β depicts the positive effect of dietary fiber in colon cancers. TGF‐β activates Smad3, after binding to its receptors on the gut epithelial cells. Butyrate also affects the gut epithelial cells and increases the Smad3 expression.^101^ Smad2 and Smad, part of the receptor‐regulated Smads (R‐Smad) family, form TGF‐β receptors substrates. Once phosphorylated, Smad2 and Smad3 interact with Smad4 interceding nuclear translocation. The Smad complex in the nucleus regulates the expression of targeted genes.^102^

## 4 CIRCULATING VERSUS FECAL SCFA

Fecal SCFAs are widely used as an indicator of microbial fermentation despite the fact that they do not accurately reflect the in vivo colonic fermentation, due to nearly 95% of colonic SCFA being absorbed and only 5% being drained away through feces.^10^ Müller et al. (2019) reported that circulating, but not fecal, SCFAs are associated to circulating GLP‐1 concentrations, peripheral insulin sensitivity and whole‐body lipolysis. This study highlighted that while circulating SCFA are directly linked to metabolic health parameters fecal SCFA do not see to have a similar effect. Therefore, circulating SCFA could potentially be employed as a biomarker for human prebiotic/probiotic intervention studies.^10^

### 4.1 SCFA and their associations with PDAC

Short‐chain fatty acids are the end products of the bacterial fermentation.^24^ Propionate and acetate are mainly produced by Bacterioidetes whilst butyrate is produced by Firmicutes. They play a crucial role in the interaction between gut microbiota and host SCFAs and can affect the progression of different diseases such as diabetes, atherosclerosis, IBD and CRC.^103^, ^104^, ^105^, ^106^ Acetate can mitigate pancreatitis offering protection against PDAC.^107^ Acetic acid has role in improving the invasiveness of PDAC cells by stimulating the epigenetic reprogramming of mesenchymal cells to cancer‐related fibroblasts.^108^ Butyric acid can reduce the growth of cultured PDAC cells and activate differentiation.^109^ It is also known that hyaluronic acid conjugate of butyrate was cytostatic in cultured PDAC cells.^110^

Ren et al. (2017) reported that butyrate‐forming microbes' concentrations are decreased in PDAC hampering; therefore, the potential SCFAs beneficial effects.^29^ Zhou et al. (2021) highlighted that the gut microbiota of PDAC patients can impact fatty acids degradation as well as the synthesis of short‐chain fatty acids (SCFAs), especially acetate and butyrate. Furthermore, the study reported a significant reduction in butyrate concentration between PDAC samples and healthy controls.^111^

Some in vitro studies reported that butyrate and its analogs have pro‐differentiating, anti‐proliferative, pro‐apoptatic, and anti‐invasive effects in PDAC cell lines,^112^, ^113^, ^114^ as well as function as histone deacetylase (HDAC) inhibitors possessing anti‐cancer and anti‐inflamatory properties as well as an anti‐fibrogenic action.^114^, ^115^

### 4.2 Diet and SCFAs in PDAC

Diet is the major component to the variation in gut microbiota, which in turn affect disease susceptibility,^116^ directly affecting functional changes that accompany particular syndromes or diseases.^116^, ^117^, ^118^

Diet‐driven changes in microbial diversity cause variations in SCFAs and future studies are necessary to understand the long term effects of these variations. In a recent study mice fed with low fiber intake resulted in depletion of butyrate production, directly disrupting gut microbial diversity, causing systemic inflammation and death due to necrotizing pancreatitis.^119^ Recently Hendifar et al. (2022) characterized the stool microbiome composition in patients with advanced PDAC who received enteral feeding for the treatment of cachexia. Almost 80% of the PDAC patients develop cachexia along disease period. A unique relationship was identified between the gut microbiome and treatment of cachexia with enteral feeding in advanced PDAC patients. Modulating the stool microbiome can be an interventional strategy to alleviate PDAC cachexia.^120^

The SCFAs are key for adjusting immune tolerance, improving gut barrier junctions and intestinal purge.^116^ Appropriate SCFA concentrations are necessary to ensure healthy metabolism and prevent disease.^11^, ^121^ Moreover, SCFAs also influence the function and the metabolism of peripheral tissues offering emerging evidence of their potential role important disease metabolic biomarkers.^11^

### 4.3 Effect of SCFAs on signaling pathways

Dietary fiber intake is an important contributor to gut health decreasing the risk of colorectal cancer. It further enhances TFG‐β signaling and growth inhibition in the gut. Cao et al. (2011) reported that butyrate enhances TFG‐β signaling in rat intestinal epithelial cells (RIE‐1) reporting that chow enriched with dietary fiber pectin resulted in increased Smad3 levels in the gut. Moreover, cells treated with either TFG‐β or butyrate alone exhibited reduced growth as well as induced cell cycle arrest. When the cells were treated with a combination of TFG‐β and butyrate, cell cycle arrest was induced, RIE‐1 cell apoptosis as well as Id2 and Id3 level reduction.^101^ Martin‐Gallausiaux et al. (2018) screened bacterial supernatants, derived from 120 commensal species on a TFG‐β_1_ system, reporting that butyrate, the main microbiota metabolite, induces TFG‐β_1_ expression in human intestinal epithelial cell line HT‐29.^122^

Farrow et al. (2003) reported that sodium butyrate causes differentiation in transformed cells but its effect on integrin expression is not known. This study determined the levels of integrin expression in pancreatic cancer cells and investigated the effect of sodium butyrate on integrin expression reporting that sodium butyrate reduces the expression of β4 integrin in pancreatic cancer cells as well as identified that β4 expression is higher in more aggressive pancreatic cancer cells. Sodium butyrate inhibits β4 expression and invasion potentially forming an innovative strategy for inhibiting pancreatic cancer invasion and improving pancreatic cancer prognosis.^123^

The genes as well as the pathways implicated in PDAC regulation are listed in Table 1.

**TABLE 1:** Genes and associated pathways impacted or regulated in the PDAC

Genes associated with PDAC	Pathway involved	Linked with SCFA	Reference
K‐RAS	1. RAF/ERK pathway 2. Phosphoinositide 3‐kinase (P13K) pathway 3. Ra1GDS pathway 4. NF‐κB		^124^
			^125^
			^126^
TFG‐β		Butyrate enhances TFG‐β signaling in rat intestinal epithelial cells.	^101^
		Butyrate induce TFG‐β_1_ expression in human intestinal epithelial cell line HT‐29.	^122^
NF‐κB	P53 (NF‐κB downregulates p53 expression)	Butyrate can inhibit NF‐kB activation in human macrophages and epithelial cells	^127^
			^128^
HDACs inhibitors	anti‐inflammatory agents	SCFAs are natural HDACs inhibitors, facilitating expressions of anti‐inflammatory genes in the immune cell	^104^
			^129^
G‐protein coupled receptors (GPCRs)	NF‐κB signaling pathway	SCFAs could activate GPR41 and GPR43 in intestinal epithelial cells, leading to transmission of mitogen‐activated protein kinase signaling, and rapid secretion of chemokines and cytokines	^130^
			^131^
		GPR87 enhanced pancreatic cancer aggressiveness by activating NF‐κB signaling pathway	^132^
Insulin‐like growth factor binding proteins (IGFBPs)	They modulate the actions of IGFs on cell proliferation and differentiation	short‐chain fatty acids regulate the secretion of IGFBPs by intestinal epithelial cells	^133^

### 4.4 Predictive markers for early detection of PDAC

There is a lack of early PDAC detection approaches due to the multiple complex interactions between microbiome and the host. The host metabolic pathways, affected by gut microbiota, is key in cancer progression on top of gut microbial dysbiosis.^134^ Mendez et al. (2020) analyzed gut microbiome and its metabolic products in a PDAC mouse models reporting that microbial metabolites can be used in patients for early pancreatic cancer detection. At very early time points of tumorigenesis, no detectable pancreatic tumors appear in KPC mice but histological pancreatic changes are denoted coinciding gut microbial population changes. Upon examining multiple bacterial species, major microbial metabolites, involved in the progression and development of PDAC tumors, are integral to polyamine metabolism. Furthermore, PDAC patients' serum samples polyamine concentrations are increased. Similarly, serum polyamine levels in KPC mice are also increased in line with tumor progression from PanINs to PDAC. Therefore, besides the gut microbial flora, microbial metabolites should be analyzed for the detection of cancer at early stages and in particular polyamines that form potential biomarkers for the PDAC detection. Moreover, when the tumor progressed in mice (4‐month sample), *Lactobacillus reuteri* was detected which was not detected at earlier ages correlating *Lactobacillus reuteri* with polyamine metabolism.^135^

## 5 DISCUSSION

### 5.1 Challenges in the microbiome research for PDAC

One of the main challenges in microbiome research lies with the poor study reproducibility as well as results inconsistencies primarily attributed to the inherent variabilities in the computational and experimental workflows.^136^, ^137^ For example, multiple PDAC studies reported variable results between the saliva microbiota profiles of PDAC patients compared to the healthy controls.^30^, ^138^, ^139^, ^140^ These discrepancies^141^ could be the result of various factors, including study sizes, study designs, sampling methods, DNA extraction methods,^142^, ^143^ patient comorbidities, patient ethnicity, dietary intake, geographic location, primers used for sequencing and statistical analysis.^136^

### 5.2 Study design and selection of the cohort

A study design is crucial for obtaining accurate and meaningful results in microbiome studies.^137^, ^144^ The environmental influence on microbiome diversity renders longitudinal study approaches preferable over cross‐sectional studies since the former are better suited to control confounding effects,^145^ albeit only a handful of well‐founded downstream analyses for such longitudinal studies have been carried out to date.^145^, ^146^

A good study design can sometimes address some study limitations, such as limited resources, small sample size, and time restrictions.^147^ A good design is essential for minimizing spurious disease associations caused by the confounding factors,^148^ such as diet,^148^, ^149^, ^150^ medication,^151^, ^152^ season,^153^ age,^154^ gender,^148^ ethnicity,^145^ body mass index (BMI),^142^, ^155^, ^156^ as well as experiment‐related confounders, such as ones related to DNA extraction methods.^142^, ^143^ It is therefore essential that such data and information should be recorded in detail so as to enable efficient downstream analysis accounting for confounding variables.^145^, ^157^, ^158^

Another microbiome study design challenge lies with the choice of the control populations. The choice of control population enables microbiome signature discrimination which can aid the advanced patient stratification and early diagnosis.^30^ Hence, the control group of the study must be carefully selected considering a clear contrast across all heterogenous phenotypes of interest in the diseased population.^30^ Control groups, although crucial for the generation of interpretable results, they are sometimes omitted in studies due to cost reduction.^147^ Moreover, compared to other nutrition or clinical trials, microbiome studies typically necessitate controls at the experiment level as well.^144^, ^147^ Negative and positive controls can reduce variability by controlling several factors such as sampling methods, DNA extraction kits, PCR blanks and contaminations.^144^, ^147^

Finally, the frequency and the timing of sample collection from the study population should be determined.^143^, ^159^

### 5.3 Power analysis and sample size

Determining a microbiome study power analysis, essential for determining the minimum sample size to detect the effect size of scientific interest without compromising resources, such as time and resources,^160^ is still an ongoing research domain.^137^ Broad study objectives typically result in underpowered study designs for sub‐groups analysis due to insufficient sample size^147^ often resulting in spurious interpretations.^145^ A crucial characteristic of microbiome analysis lies with the variable microbial load even between the biological samples under similar conditions.^145^, ^161^ Therefore, in case of unknown or small effect sizes, identifying weak biological signals between similar samples is challenging rendering such studies unable to reflect on general populations of interest.^145^

### 5.4 Sequencing methods

Gene amplicon sequencing and whole‐genome shotgun (WGS) sequencing are the most commonly used methods to reveal microorganism diversity.^145^, ^162^ Among gene amplicon sequencing, 16S rRNA (or 16 s rDNA) is the most commonly used target to assign taxonomic classification.^163^ Moreover, its relatively short size renders it easier, and very cost‐effective in comparison to WGS, to sequence when dealing with large sample sizes.^145^, ^164^ However, unlike bacteria, pathogenic yeast and fungi gene identification targets are still not well‐defined^146^ with 16S rRNA sequencing offering a limited taxonomic resolution.^165^

On the other hand, WGS improves the accuracy of the sequencing of the mixture of microbial community DNA enabling the identification of present organisms as well as the assessment of phage and viruses even in the absence of any phylogenetic markers.^164^, ^166^ Moreover, it caters the identification of microbial organisms' functional capabilities.^164^, ^167^, ^168^

The bacterial population variability, depicted across microbiome studies, presents a major challenge for identifying universal biomarkers, potentially hindering the translatability of microbiome research. This effect is amplified by the limitation of laboratory mouse models to predict complex human physiological responses.^169^, ^170^ Therefore, both the microbiome variability and the poor inter‐species reproducibility present challenges that should be addressed for developing translational research models.

## 6 OPPORTUNITIES

Targeting specific microbiomes and understanding their role in the specific cancer types can act as an early intervention. Moreover, diet plays a significant role in modification of the gut microbes both on a short and a long‐term scale on human health, which implies that diet manipulation will impact gut microbes' composition that potentially can be used as therapeutics approach. Such an approach would potentially include prebiotic or probiotic substances to ensure gut microbiome diversity.

## 7 CONCLUSIONS

The SCFAs and their associated pathways are dysregulated in PDAC. For example, the KRAS genes are involved in 3 downstream pathways all of which have been implicated in PDAC tumorigenesis. For example, butyrate has an effect on NF‐κB activation and there is potentially an association between butyrate and K‐RAS pathway. Moreover, NF‐kB downregulates p53 expression, and upon butyrate‐induced inhibition of NF‐κB activation the p53 expression is affected. The precise targeting of these pathways and SCFAs in PDAC patients will add novel information about PDAC treatment. Diet induced increase of SCFA abundance can directly affect the tumor microenvironment as well as the downregulation of inflammation.

Due to poor prognosis and high potential for early metastasis, biomarkers are necessary for the PDAC early detection and diagnosis. Microbiome analyses offer the tantalizing potential of forming such non‐invasive diagnostic, symptomatic and predictive biomarkers. Determining the composition of gut microbiota in PDAC patients will also cater the improved survival prediction as well as novel potential personalized treatment approaches.

## AUTHOR CONTRIBUTIONS

**Hülya Yılmaz Temel:** Data curation (lead); investigation (equal); visualization (equal); writing – original draft (equal); writing – review and editing (equal). **Öznur Kaymak:** Data curation (equal); formal analysis (equal); visualization (equal); writing – original draft (equal); writing – review and editing (equal). **Seren Kaplan:** Data curation (equal); formal analysis (equal); investigation (equal); writing – original draft (equal); writing – review and editing (equal). **Basak Bahcivanci:** Data curation (equal); investigation (equal); writing – original draft (equal); writing – review and editing (equal). **Georgios V. Gkoutos:** Funding acquisition (lead); methodology (equal); supervision (lead); writing – original draft (equal); writing – review and editing (equal). **Animesh Acharjee:** Conceptualization (lead); data curation (supporting); formal analysis (supporting); investigation (lead); methodology (lead); project administration (lead); supervision (lead); validation (equal); writing – original draft (equal); writing – review and editing (equal).

## FUNDING INFORMATION

This work was funded by MRC Health Data Research UK (HDRUK/CFC/01), an initiative funded by UK Research and Innovation, Department of Health and Social Care (England) and the devolved administrations, and leading medical research charities. The views expressed in this publication are those of the authors and not necessarily those of the NHS, the National Institute for Health Research, the Medical Research Council or the Department of Health. Animesh Acharjee and Georgios V. Gkoutos also acknowledge support from the NIHR Birmingham SRMRC, Nanocommons H2020‐EU (731032) and MAESTRIA (Grant agreement ID 965286).

## CONFLICTS OF INTEREST

The authors have no conflicts of interest to declare. All co‐authors have seen and agree with the contents of the manuscript and there is no financial interest to report.

## ETHICAL APPROVAL STATEMENT

The current study is exempt from ethical approval.

## Supporting information


Appendix S1

## Data Availability

NA
